# Experimental demonstration of the possible role of *Acanthamoeba polyphaga* in the infection and disease progression in Buruli Ulcer (BU) using ICR mice

**DOI:** 10.1371/journal.pone.0172843

**Published:** 2017-03-22

**Authors:** Bright K. Azumah, Phyllis G. Addo, Alfred Dodoo, Gordon Awandare, Lydia Mosi, Daniel A. Boakye, Michael D. Wilson

**Affiliations:** 1 Department of Animal Experimentation, Noguchi Memorial Institute for Medical Research, University of Ghana, Legon, Accra, Ghana; 2 Department of Biochemistry, Cell and Molecular Biology, University of Ghana, Legon, Accra, Ghana; 3 Department of Electron Microscopy and Histopathology, Noguchi Memorial Institute for Medical Research, University of Ghana, Legon, Accra, Ghana; 4 West African Centre for Cell Biology of Infectious Pathogens, Department of Biochemistry, Cell and Molecular Biology, University of Ghana, Legon, Accra, Ghana; 5 Department of Parasitology, Noguchi Memorial Institute for Medical Research, University of Ghana, Legon, Accra, Ghana; University of Groningen, University Medical Center Groningen, NETHERLANDS

## Abstract

The transmission of Buruli ulcer (BU), caused by *Mycobacterium ulcerans* (*MU*), remains puzzling although a number of hypothesis including through bites of infected aquatic insects have been proposed. We report the results of experiments using ICR mice that give credence to our hypothesis that *Acanthamoeba* species may play a role in BU transmission. We cocultured *MU* N2 and *MU* 1615 which expresses red fluorescent protein (RFP) and *Acanthamoeba polyphaga* (*AP*), and confirmed infected *AP* by Ziehl-Neelsen (ZN) staining. We tested for viability of *MU* inside *AP* and observed strong RFP signals inside both trophozoites and cysts after 3 and 42 days of coculturing respectively. ICR mice were topically treated, either on shaved intact or shaved pinpricked rumps, with one of the following; *MU* N2 only (2.25 x 10^6^ colony forming units [CFU] / ml), *MU* N2:*AP* coculture (2.96 x 10^4^ CFU: 1.6 x 10^6^ cells/ml), *AP* only (1.6 x 10^6^ cells/ml), PYG medium and sterile distilled water. Both *MU* N2 only and *MU* N2:*AP* elicited reddening on day (D) 31; edema on D 45 and D 44 respectively, and ulcers on D 49 at pinpricked sites only. To ascertain infectivity and pathogenicity of *MU* N2 only and *MU* N2:*AP*, and compare their virulence, the standard mouse footpad inoculation method was used. *MU* N2:*AP* elicited reddening in footpads by D 3 compared to D 14 with *MU* N2 only of the same dose of *MU* N2 (2.96 x 10^4^ CFU). ZN-stained *MU* were observed in both thin sectioned and homogenized lesions, and aspirates from infected sites. Viable *MU* N2 were recovered from cultures of the homogenates and aspirates. This study demonstrates in ICR mice *MU* transmission via passive infection, and shows that punctures in the skin are prerequisite for infection, and that coculturing of *MU* with *AP* enhances pathogenesis.

## Introduction

Buruli ulcer (BU), a necrotic skin disease caused by *Mycobacterium ulcerans*, is the third most prevalent mycobacterial infection after tuberculosis and leprosy [1]. It has been reported in West Africa, Central Africa, East Africa, Australia, China, South America and Japan with West Africa being the most endemic region [2].

Although the associated risk factors have been adequately studied [2,3], the mode of transmission still remains poorly understood [2]. Two main transmission hypotheses have been expounded; direct inoculation into broken skin through contact with contaminated environmental sources [2,4] and vector-mediated transmission [4–7].

Based on the detection of *M*. *ulcerans* DNA in the environment, many agents have been speculated as possible reservoirs [2] and this has given support to the proposition that contact with environmental reservoirs is the source of transmission. However analysis of the *M*. *ulcerans* genome and pathogenic mechanisms have revealed genome reduction and intracellular niche specialization in the environment [8–10] thus indicating that biological reservoirs such as amoebae may also be likely candidates.

It has been demonstrated in laboratory studies that biting aquatic bugs (Naucoridae) fed on *M*. *ulcerans*-infested grub, could transmit *M*. *ulcerans* through bites and cause BU lesions in mice [7]. In Australia, mosquitoes have also been implicated as possible insect vectors because *M*. *ulcerans* DNA has been detected in lysates of pooled mosquitoes. Also the *M*. *ulcerans* DNA positivity rate of sampled mosquitoes correlated with BU endemicity in local communities [11]. Additionally, a laboratory-based study showed that *M*. *ulcerans* DNA was found to persist in three successive instars of mosquito [12]. A recent experimental mouse-tail infection model has shown that *Anopheles notoscriptus*, could transmit *M*. *ulcerans* to mice through bites and cause BU [13].

Free living amoebae (FLA) have been reported severally in literature as possible reservoirs of pathogenic mycobacteria [14–20]. The difficulty in implicating an arthropod vector for the transmission of leprosy, and the intracellular-niche-requiring character of *M*. *leprae* led to the demonstration that *Acanthamoeba* spp could successfully maintain viable *M*. *leprae* intracellularly [17]. This implicates *Acanthamoeba* spp as an important reservoir in the transmission of leprosy in nature. Similarly, we had earlier posited that *Acanthamoeba* spp may play an important role in BU transmission but did not support it with data [21]. However a study by Gryseels et al. [22] undermined the potential role of *Acanthamoeba* in BU transmission in the environment. The current study provides evidence, albeit in a laboratory model, in support of the hypothesis that *Acanthamoeba* spp may play a role in BU transmission. Furthermore, we also investigated whether *Acanthamoeba polyphaga* would enhance the virulence of *M*. *ulcerans* as reported for *M*. *leprae* [19] and *M*. *avium* [20].

Studies have demonstrated that injection of *M*. *ulcerans* into the footpad of mice [23], skin of grasscutters [24] and guinea pigs [25] results in BU, but topical application of *M*. *ulcerans* on the abraded skin of the same guinea pigs did not lead to BU [25], suggesting that deeper dermal inoculation is required for transmission. Here, we show in an ICR mouse model that passive inoculation of naked *M*. *ulcerans* via contact with punctured skin could result in BU. We also show here for the first time that the mouse model could present undermined ulcer, which is the hallmark of BU. Finally, our study demonstrates for the first time that *A*. *polyphaga* cocultured with *M*. *ulcerans* causes BU. Our study also demonstrates that coculturing *A*. *polyphaga* with *M*. *ulcerans* enhances BU pathogenesis.

## Methods

### Ethical considerations

The Scientific and Technical Committee of Noguchi Memorial Institute for Medical Research (NMIMR) approved the study. The study protocols and procedures received approval from the NMIMR Institutional Animal Care and Use Committee (NIACUC#: 2014-01-1N). The NIACUC is governed by the Public Health Service (PHS), Animal Welfare Act (AWA) and the United States Department of Agriculture and is guided by the U.S. Government Principles for the Utilization and Care of Vertebrate Animals Used in Testing, Research and Training.

Standard hygienic practices of laboratory animal maintenance were ensured. Animal usage was in accordance with Applied Research Ethics National Association and the institutional guidelines on animal research.

### Animals and maintenance

Male and female outbred ICR mice produced in the Department of Animal Experimentation of the Noguchi Memorial Institute for Medical Research were used for the experiments. Forty-eight (48) mice were put into 16 groups of three mice per cage. The mice were maintained in a level two animal containment facility with a 12–12 hour light-dark cycle automatic lighting system, an ambient temperature of 23–25°C and relative humidity of 55–65%. The mice were fed daily on autoclaved feed pellets and water *ad libitum*.

### Maintenance of *Mycobacterium ulcerans* and *A*. *polyphaga* cultures

The *Mycobacterium ulcerans* N2 strain used in this study was isolated from biopsy of a BU patient who had to undergo reparative surgical treatment at Nsawam General Hospital, Ghana in 2007. Our laboratory served as a reference laboratory that undertook confirmatory tests for BU cases that year. The *M*. *ulcerans* N2 strain was identified as an IS*2404* positive organism and isolates were cultured on Lӧwenstein-Jensen (LJ) plates at 32°C for 8 weeks. The *M*. *ulcerans* 1615::RFP and *A*. *polyphaga* 1501/3b used in this study were kind donations from Heather Williamson formerly of the University of Tennessee, USA to Lydia Mosi. The *A*. *polyphaga* trophozoites were grown in PYG medium in 25 cm^2^ culture flask at 30°C.

### Preparation of *M*. *ulcerans* suspension

*Mycobacterium ulcerans* (all strains) suspension [(2.25 ± 0.05) x 10^6^ CFU/ml) was prepared from eight-week-old axenic cultures. Briefly, colonies of *M*. *ulcerans* grown on LJ slants were scraped off and introduced into 1ml sterile double distilled water in sterile universal culture bottle that contained sterile glass beads. The tightly closed bottle was placed on ice for 2 minutes and then vortexed vigorously for 20 seconds to break up the clumps of *M*. *ulcerans*. The cycle was repeated with intermittent addition of distilled water; the suspension was allowed to settle and thereafter the supernatant was decanted into sterile universal bottle. The turbidity of the supernatant was matched with freshly prepared McFarland (McF) 5 nephelometric standard and adjustments made by intermittent addition of sterile distilled water. The adjusted supernatant was used within an hour. To determine the CFU/ml of the McF 5 *M*. *ulcerans* suspension, 100 μl each of 1 in 10 dilutions (10^−1^ to 10^−9^) of McF 5 *M*. *ulcerans* suspension was inoculated on LJ slants in triplicates and incubated at 32°C for eight weeks. The average CFU/ml was determined for the 1 in 100 dilution inocula on three LJ slants.

### Coculture of *A*. *polyphaga* trophozoites and *M*. *ulcerans*

*Acanthamoeba polyphaga* trophozoites were cultured in PYG medium at 30°C until confluence reached about 70%. The viable cell density was determined to be 1.6 ± 0.1 x 10^6^ cells/ml. This was done by staining cells with 0.4% Trypan Blue solution (Gibco, UK) in a 1:4 ratio, and counting viable cells using a Neubauer improved Hemocytometer (Blaubrand, Germany).

An aliquot of 200μl *M*. *ulcerans* suspension [(2.25 ± 0.05) x 10^6^ CFU/ml] was added to 15 ml of PYG medium containing 1.6 ± 0.1 x 10^6^ cells/ml of *A*. *polyphaga* trophozoites in 25 cm^2^ culture flask. The coculture was incubated at 30°C with daily observation for three days for the animal infection studies, and 42 days for viability studies using red fluorescent protein expression by *M*. *ulcerans* 1615::RFP in trophozoites and cysts respectively. Culturing for 42 days without changing media stresses the trophozoites through nutrient depletion and thus makes them encyst.

### Direct examination of *M*. *ulcerans* viability using fluorescence from *M*. *ulcerans*::RFP inside trophozoites and cysts of *A*. *polyphaga*

*Mycobacterium ulcerans*: *A*. *polyphaga* coculture (ratio = 2.96 x 10^4^ CFU/ml: 1.6 x 10^6^ cells/ml) was incubated for 3 days and 42 days without changing growth medium. For the three day culture, the medium was decanted and washed twice (3000 rpm for 5 minutes at 25°C) with freshly prepared PYG medium. The washed trophozoites were finally detached from the walls of the flask using 0.25% Trypsin—EDTA (1X) solution (Gibco, UK). Briefly, 1ml of the Trypsin solution was added to adhered trophozoites in the flask and incubated at 30°C for 5 minutes. Thereafter, 15ml of PYG medium was added and the flask was tapped gently to suspend the cells in solution. The cell suspension was then centrifuged at 3000rpm for 5 minutes at 25°C. The supernatant was decanted and the pelleted cells suspended in 5 ml of freshly prepared PYG medium and then 1 ml aliquots dispensed onto sterile glass cover slides. Slides were incubated for 10 minutes in a humid chamber and thereafter observed through the red channel of a fluorescent microscope (Olympus VX41, Japan). For the cysts, the 42-day old *M*. *ulcerans*: *A*. *polyphaga* coculture was harvested by centrifuging at 3000 rpm for 5 minutes at 25°C. Cysts were washed twice with sterile distilled water as described and finally centrifuged to concentrate cell pellets. The cells were then applied onto slides and similarly observed under a fluorescent microscope.

### Coculture preparation for animal infection studies

After incubation, the medium was decanted and the adhered trophozoites were washed three times with freshly prepared PYG medium. Supernatant from the last wash was inoculated on LJ slants for growth of *M*. *ulcerans* and also examined for the presence of extracellular *M*. *ulcerans* using Ziehl-Neelsen (ZN) acid-fast staining method. Extracellular mycobacteria were not seen in supernatant, neither was *M*. *ulcerans* growth observed on LJ after 10 weeks of incubation. The washed trophozoites were finally detached from the walls of the flask using 0.25% Trypsin—EDTA (1X) solution (Gibco, UK) as described above. After centrifugation, the cell pellet was suspended in 5 ml of freshly prepared PYG medium. This was used immediately for the infection experiments. An aliquot of 100μl of the cell suspension was ZN-stained and examined under the microscope (1000X) to confirm the internalization of *M*. *ulcerans* in *A*. *polyphaga* trophozoites. Additionally, 2 ml aliquot of the cell suspension was treated with 2ml of 0.5% sterile SDS solution for 5 minutes to lyse the trophozoites and then washed three times with sterile distilled water by centrifuging at 3000 rpm for 5 minutes. The lysate was again reconstituted in 2ml of sterile distilled water. An aliquot of 1 ml of the cell lysate was then inoculated on LJ slants and incubated at 32°C to confirm growth of intracellular *M*. *ulcerans*.

### Animal infection experiments

Forty-eight (48) ICR mice were placed under two treatment groups; A and B. In group A, 9 mice divided equally into three groups, were shaved on their rumps and the area disinfected with 70% ethanol. The rumps were then superficially pinpricked (10 pinpricks at a depth of about 1.5mm) using a sterile 27-gauge TERUMO needle (TERUMO Corporation, Japan) over an area of 1cm^2^. The three groups of mice (3 mice each) were then infected by topical application of 50 μl of each of the following inocula: i) 2.25 x 10^6^ CFU/ml *M*. *ulcerans*, ii) *M*. *ulcerans*: *A*. *polyphaga* (ratio = 2.96 x 10^4^ CFU/ml: 1.6 x 10^6^ cells/ml) and iii) 1.6 x 10^6^ cells/ml *A*. *polyphaga*. Three other groups of mice were shaved on their rumps but not bruised. The skins of these groups were similarly treated as described above. Negative control groups (n = 12) were shaved on their rumps and the skin was either left intact or pinpricked. The intact and the pinpricked skins were treated with either distilled water or PYG medium or left untreated.

In the last treatment group (B), the right footpads of 12 mice, divided equally into four groups, were disinfected with 70% ethanol and injected with 50 μl suspensions of either; i) 2.25 x 10^6^ CFU/ml *M*. *ulcerans*, or ii) 2.96 x 10^4^ CFU/ml *M*. *ulcerans*, or iii) *M*. *ulcerans*: *A*. *polyphaga* (ratio = 2.96 x 10^4^ CFU/ml: 1.6 x 10^6^ cells/ml), or iv) 1.6 x 10^6^ cells/ml *A*. *polyphaga*. In the negative control group of 6 mice, divided into two equal groups, the right footpads were injected with either 50 μl of distilled water or PYG medium. The mice were monitored daily for development of BU lesions. Mice from both *M*. *ulcerans* only and *M*. *ulcerans*: *A*. *polyphaga* treatment groups that had developed full-blown ulcers were euthanized by injecting intraperitoneally with an overdose of pentobarbital (200 mg/kg IP). The ulcerated skin tissues were surgically removed and immediately immersed in a 10x volume of neutral 10% formalin for subsequent histological processing.

### Isolation of *M*. *ulcerans* from lesions and microbiological analysis

Samples of lesions from live and dead mice were taken and examined for the presence of *M*. *ulcerans*. In the case of live mice, fine needle aspirates (FNA) of lesions were taken aseptically. A portion of the sample was inoculated on LJ slants and incubated for 8 weeks at 32°C for *M*. *ulcerans* growth, while the remaining portion was smeared on glass slides and ZN-stained for detection of AFBs. Lesions from dead mice were excised, homogenized and decontaminated using the Petroff’s decontamination method [26]. Briefly, tissues were homogenized in 2ml sterile distilled water by grinding in a sterile mortar and mixed with 2ml of 4% NaOH to digest for 20 minutes with intermittent agitation. The suspension was then mixed with 2ml of 1% HCl containing Safranin red to neutralize the basic homogenate. The suspension was centrifuged at 3000 rpm for 20 minutes at 25°C. The pellet was washed twice with 10ml distilled water and centrifuged as described above and finally suspended in 1ml distilled water. One hundred microliters of the final suspension was inoculated on LJ slants and incubated at 32°C for *M*. *ulcerans* growth. The remaining suspension was also ZN-stained for AFB detection.

### Histological examination of ulcerated skin tissues

The formalin preserved tissues obtained from the euthanized mice were embedded in wax and thereafter thin sections of 5 microns thickness were cut using a microtome (Bright 5040, England). The sections were then fixed on sterile slides and ZN-stained.

## Results

### Internalization and viability of *M*. *ulcerans* (acid-fast bacilli) within *A*. *polyphaga* trophozoites and cysts

**Fig 1:**
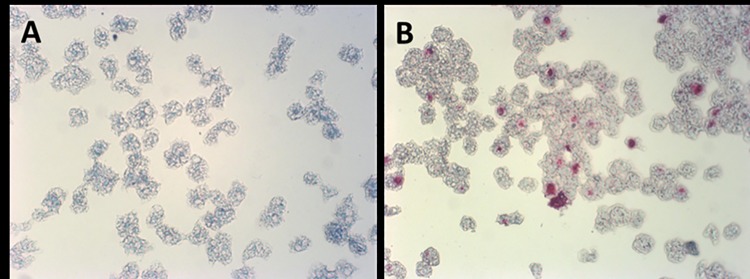
Visualization of *M*. *ulcerans* within *A*. *polyphaga* trophozoites. Panel A shows ZN stain of *A*. *polyphaga* trophozoites. No acid-fast particle was seen in the axenic culture of *A*. *polyphaga* trophozoites. Panel B shows a ZN stain of *A*. *polyphaga* trophozoites infected with *M*. *ulcerans*. *A*. *polyphaga* trophozoites stained bluish and *M*. *ulcerans* stained reddish- pink (acid-fast particles). Acid fast particles denoting the presence of *M*. *ulcerans* can be seen inside trophozoites of *A*. *polyphaga* infected with *M*. *ulcerans*. The average rate of infection was 47% ± 8.6 (SD) (average counts in 5 fields randomly selected under 200x magnification).

To determine whether *A*. *polyphaga* could successfully internalize and harbor *M*. *ulcerans*, axenic culture of *A*. *polyphaga* was cocultured with *M*. *ulcerans* for three days. No acid-fast particles were observed in axenic cultures of *A*. *polyphaga* when ZN stained [stained bluish] (Fig 1A). Acid-fast bacilli (stained reddish-pink) were consistently found internalized in trophozoites of *M*. *ulcerans*-infected *A*. *polyphaga* 3 days post-culture (Fig 1B). Profuse growth of *M*. *ulcerans* colonies were also observed after incubating *M*. *ulcerans*-infected *A*. *polyphaga* cell lysates on LJ slants for 8 weeks.

**Fig 2:**
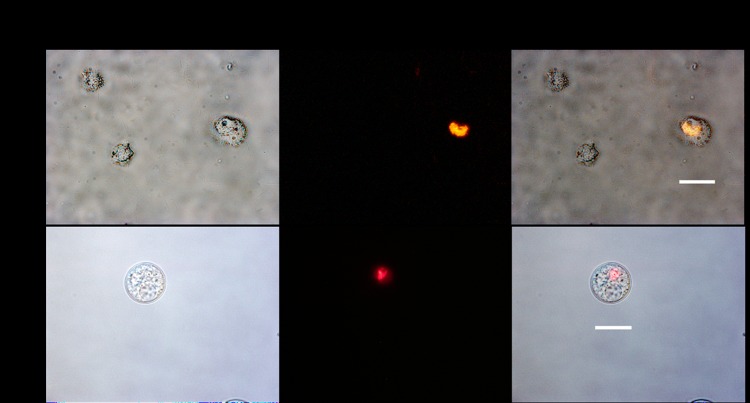
Detection of strong RFP signals of *M*. *ulcerans* 1615::RFP inside trophozoites and cyst of *A*. *polyphaga*. RFP signals were detected in trophozoites (3 days coculture) and also cyst (42 days coculture). Scale bar = 20μm.

Strong red fluorescence from expressed red fluorescent proteins of *M*. *ulcerans* 1615::RFP were intracellularly localized in *A*. *polyphaga* trophozoites and cysts, three and 42 days respectively of coculturing (Fig 2).

### Passive infection of mice with *M*. *ulcerans* only and *M*. *ulcerans*: *A*. *polyphaga* coculture

Previous studies have shown that *M*. *ulcerans* alone, without hypodermal injection, was incapable of spontaneously infecting lacerated skins of guinea pigs [25] by passive inoculation, therefore it was of interest to determine if *A*. *polyphaga* cocultured with *M*. *ulcerans* could confer passive infection of broken skin.

**Fig 3:**
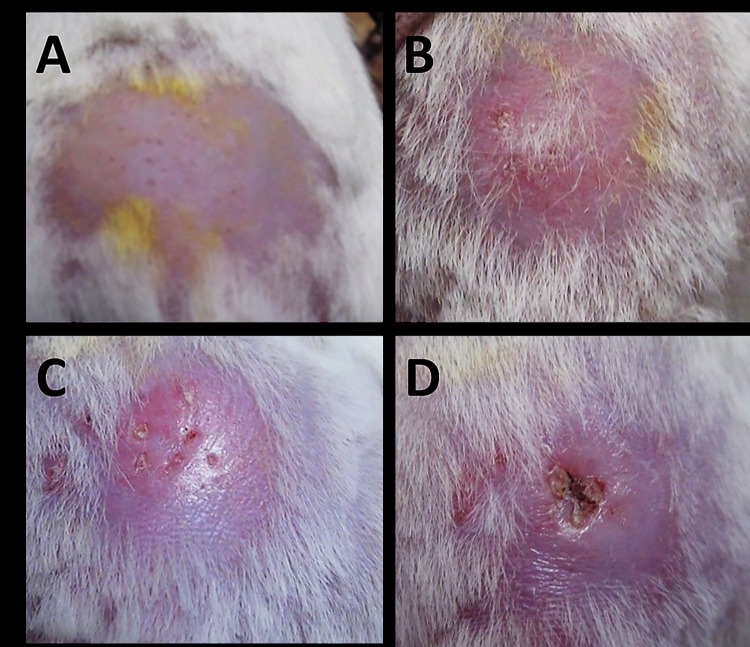
The progressive development of BU in mouse topically treated with *M*. *ulcerans*-infected *A*. *polyphaga* at the punctured skin of the rump (lower back). Panel A shows site of inoculation 1 day post-inoculation (dpi). Panel B shows inflammation (erythema) at site of inoculation 31 dpi. Panel C shows an edema at site of inoculation 44 dpi. Panel D shows ulcer at the site of inoculation 49 dpi. Photograph is representative of group (n = 3).

Progressive development of BU lesions were observed in mice when the punctured skins of the rump (Fig 3A) were topically treated with *A*. *polyphaga*-infected *M*. *ulcerans*. Inflammation (erythema) was observed at the punctured sites 31 days post inoculation (dpi) (Fig 3B), followed by edema on 44 dpi (Fig 3C) and ulcers on 49 dpi (Fig 3D). Fine needle aspirate (FNA) samples from the ulcers were all AFB positive.

Similarly, topical application of 2.25 x 10^6^ CFU/ml of *M*. *ulcerans* on punctured skin also elicited BU lesions (S1 Fig) with the same characteristics as that seen with *M*. *ulcerans*-infected *A*. *polyphaga*. This also elicited visible inflammation at the inoculation sites on 31 dpi (Table 1). However, in mice with intact shaved skins, none of the treatments elicited any noticeable pathological response even after five months of monitoring.

**Table 1:** The progression of BU signs in the various treatment groups post-inoculation.

Experiments	Earliest time (days) of visible signs post-inoculation (respondents/number of mice)			AFB in fine needle aspirate (FNA) from ulcer	*M*. *ulcerans* culture from FNA and from ulcer
Infection by topical application of inoculum on punctured skin	Inflammation	Edema	Ulcer		
50 μl of *M*. *ulcerans* (2.25 x 10^6^ CFU/ml), positive control **(Group 1, n = 3)**	31 (3/3)	45 (3/3)	49 (3/3)	Yes (3/3)	Yes (3/3)
50 μl of *A*. *polyphaga* (1.6 x 10^6^ cells/ml) infected with *M*. *ulcerans* (2.96 x 10^4^ CFU / ml) **(Group 2, n = 3)**	31 (3/3)	44 (3/3)	49 (3/3)	Yes (3/3)	Yes (3/3)
50 μl of *A*. *polyphaga* trophozoites (1.6 x 10^6^ cells/ml) **(Group 3, n = 3)**	None (3/3)	None	None	Not applicable (N/A)	N/A
50 μl of PYG **(Group 4, n = 3)**	None (3/3)	None	None	N/A	N/A
50 μl of distilled water **(Group 5, n = 3)**	None (3/3)	None	None	N/A	N/A
**Infection by injection into footpad**					
50 μl of *M*. *ulcerans* (2.25 x 10^6^ CFU / ml,) positive control **(Group 4, n = 3)**	1 (3/3)	1 (3/3)	21 (3/3)	Yes (3/3)	Yes (3/3)
50 μl of *A*. *polyphaga* (1.6 x 10^6^ cells/ml) infected with *M*. *ulcerans* (2.96 x 10^4^ CFU / ml)) **(Group 5, n = 3)**	3 (3/3)*	7 (3/3)	25 (3/3)	Yes (3/3)	Yes (3/3)
50 μl of *M*. *ulcerans* (2.96 x 10^4^ CFU / ml) **(Group 6, n = 3)**	14 (3/3)	31 (3/3)	51 (3/3)	Yes (3/3)	Yes (3/3)
50 μl of PYG **(Group 4, n = 3)**	None (3/3)	None	None	N/A	N/A
50 μl of distilled water **(Group 5, n = 3)**	None (3/3)	None	None	N/A	N/A

AFB = Acid fast bacilli, FNA = Fine needle aspirate

### Effect of coculturing *A*. *polyphaga* with *M*. *ulcerans* on pathogenesis of BU

The virulence of some mycobacteria has been reported to be enhanced when cocultured with *Acanthamoeba* spp [16,19,20]. We therefore tested the hypotheses that coculturing *M*. *ulcerans* with *A*. *polyphaga* will enhance the pathogenesis of BU.

**Fig 4:**
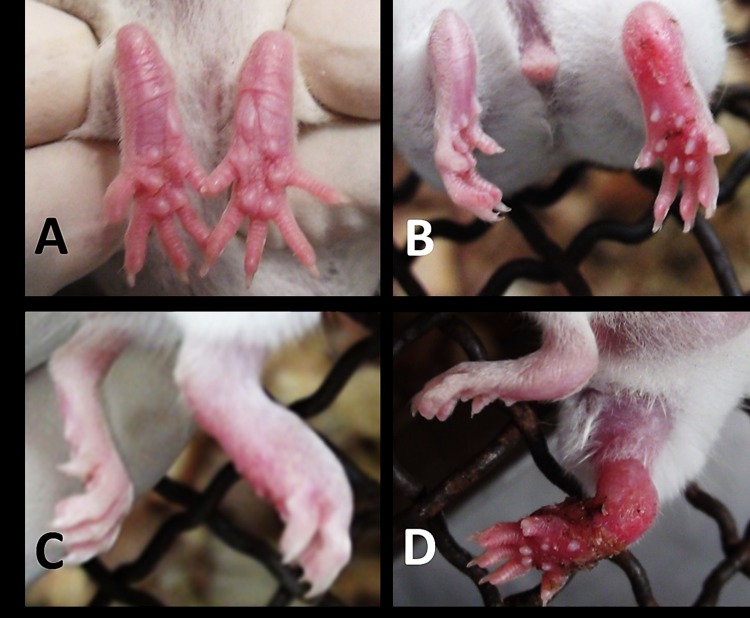
The progressive stages of development of BU in mouse injected in the right footpad with *M*. *ulcerans*-infected *A*. *polyphaga*. Panel A shows footpad 1 dpi. Panel B shows footpad with erythema 3 dpi. Panel C shows footpad with edema 7 dpi. Panel D shows swollen footpad and thigh 25 dpi. The photographs are typically representatives of each group (n = 3).

Footpads injected with *M*. *ulcerans*:*A*. *polyphaga* coculture (ratio = 2.96 x 10^4^ CFU/ml: 1.6 x 10^6^ cells/ml) showed inflammation on 3 dpi, whiles footpads injected with the same concentration of naked *M*. *ulcerans* showed inflammation 14 dpi (Table 1). Subsequent progressive signs appeared earlier for *M*. *ulcerans*:*A*. *polyphaga* than those of *M*. *ulcerans* alone of the same concentration (Table 1). However inoculation with a higher dose of naked *M*. *ulcerans* (2.25 x 10^6^ CFU/ ml) showed inflammation earlier (1 dpi) than a lower dose (2.96 x 10^4^ CFU/ ml) in coculture with *A*. *polyphaga* (3 dpi). Treatment with *A*. *polyphaga* alone at the same dose of 1.6 x 10^6^ cells/ml did not show any lesion for the entire duration of study (5 months). *M*. *ulcerans*:*A*. *polyphaga* coculture and *M*. *ulcerans* alone showed similar signs (Fig 4), while no symptom was observed in the two controls; PYG and distilled water treatments.

### Microbiological and histopathological examination of lesions

**Fig 5:**
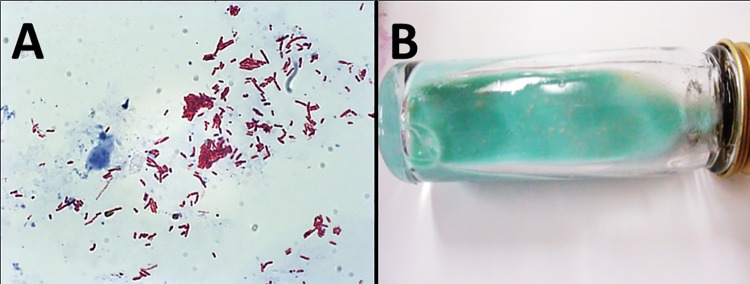
Demonstration of viable *M*. *ulcerans* in lesions. Panel A shows a ZN stain of tissue homogenate from footpad of mouse injected with *M*. *ulcerans*-infected *A*. *polyphaga*. Profuse acid-fast particles (reddish pink) were seen in tissue homogenate. Panel B shows LJ slant (green) with colonies of *M*. *ulcerans* (yellowish) recovered from tissue homogenate of the footpad of mouse injected with *M*. *ulcerans*-infected *A*. *polyphaga*.

ZN staining of the homogenates of infected footpads of mice injected with *M*. *ulcerans* alone and *M*. *ulcerans*-*A*. *polyphaga* coculture confirmed the presence of acid-fast bacilli (Fig 5A). Also, viable *M*. *ulcerans* colonies were recovered from the culture of tissue homogenates (Fig 5B).

**Fig 6:**
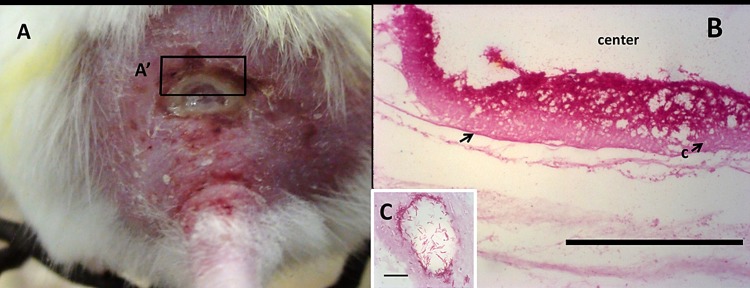
Gross morphology and ZN-stained section of the ulcer showing the distribution of acid-fast bacilli (i.e. *M*. *ulcerans*). Panel A shows undermined ulcer at the back of a mouse. Panel B shows ZN stained thin section of A´ with an arrow showing bed of acid-fast bacilli localized at the edge of the ulcer. Panel C shows ring-like clustering unit of acid fast bacilli in panel B (the scale bar measures 10 μm).

ZN stained thin sections of the ulcerated skin of mouse (Fig 6A) treated either with *M*. *ulcerans*-*A*. *polyphaga* coculture or *M*. *ulcerans* only showed presence of acid fast bacilli localized at the margins adjacent to the center of the ulcer (Fig 6B). Acid-fast bacilli appeared clustered in ring-like units (Fig 6C).

## Discussion

Several mycobacteria including *M*. *ulcerans* have been shown to survive in free living amoeba [27]. Studies by Gryseels et al. [22] and Amissah et al. [28] have also confirmed that *M*. *ulcerans* can survive in *Acanthamoeba* spps. Our study also confirms the survival of *M*. *ulcerans* 1615::RFP in trophozoites and cysts of *A*. *polyphaga* over 3 days and 42 days respectively. Cysts of *Acanthamoeba* spp have been reported to protect bacteria against extreme conditions of pH, humidity, nutrient availability, biocides and temperature [27]. The presence of viable *M*. *ulcerans* in cysts of *A*. *polyphaga* thus suggests the possibility of *A*. *polyphaga* cysts serving as protective reservoirs of *M*. *ulcerans* in unfavorable environmental conditions. Although Gryseels et al. [22] were not able to detect the presence of *M*. *ulcerans*-infected free living amoeba in the environment and further suggested their unlikely role in BU transmission, we are of the contrary view that this could not be ruled out and thus recommend further studies.

*M*. *ulcerans*-infected mosquitoes and aquatic bugs have been shown to cause BU lesions by biting intact tails of mice in controlled laboratory conditions [7,13]. Williamson et al. [25] also showed that hypodermal injection of *M*. *ulcerans* into the skin of guinea pigs was the only means by which infections could be established. BU lesions are mostly distributed specifically at the elbows and ankles, and generally at lower body extremities that are less protected by clothings [29,30]. In Cameron, women who reportedly covered their skin had less lesions on their trunks [29]. In Southeasten Australia, the odds of contracting BU was halved when insect repellents were applied and long trousers worn, both protecting the lower extremeties against insect bites [31]. Agreeably, the odds of having BU was found to be doubled for people who reported receiving mosquito bites at the lower extremeties [31]. These studies collectively support mechanical inoculation, amongst other possible means, as a possible route of BU transmission.

We have shown experimentally, and for the first time, that when both *M*. *ulcerans* alone and *A*. *polyphaga-M*. *ulcerans* coculture were applied externally on punctured skin, and not by mechanical inoculation, it resulted in infections and subsequent disease progression. Recently Jordan et al. [32] using molecular methods showed that *M*. *ulcerans* could be a contaminant on the skin. Skin abrasions happen in nature when passing through vegetation and also through normal human activities such as farming. We therefore propose that these abrasions and other sources of wounding may serve as portals of entry for *M*. *ulcerans* and *M*. *ulcerans*-infected *Acanthamoeba* spp that settle on the skin.

In this study, the skin infection caused by both naked *M*. *ulcerans* and *M*. *ulcerans*-*A*. *polyphaga* coculture progressed from an initial stage of inflammation (reddening, swelling), followed by eye-spot ulcers at the sites of infection, which progressed to become full-blown ulcers with undermined edges. The times of the appearance of signs were similar for both the higher dose of 2.25 x 10^6^ CFU/ml *M*. *ulcerans* alone and the lower dose of 2.96 x 10^4^ CFU/ml of *M*. *ulcerans* in the coculture.

The virulence of some mycobacteria has been shown to be enhanced by coculturing with *Acanthameoba* spps [16,19,20]. Based on this premise we hypothesized that *A*. *polyphaga* in coculture with *M*. *ulcerans*, will enhance the pathogenesis of BU. To test the *A*. *polyphaga*-enhanced BU pathogenicity hypothesis, we injected into mice footpads the same dose (2.96 x 10^4^ CFU/ml) of *M*. *ulcerans* alone, and *M*. *ulcerans*:*A*. *polyphaga* coculture, and observed a marked difference in times of appearance of signs. *M*. *ulcerans* alone elicited inflammation (reddening) of the footpads 14 dpi whiles for *M*. *ulcerans*-*A*. *polyphaga* coculture, inflammation appeared 3 dpi. The corresponding times for edema were 31 and 7, and for the development of ulcers were 52 and 25 dpi respectively. Altogether these results suggest that *A*. *polyphaga* enhances the virulence of *M*. *ulcerans* when cocultured. Therefore, this study has for the first time demonstrated that the pathogenicity of BU is enhanced when *M*. *ulcerans* is cocultured with *A*. *polyphaga*. Our finding is consistent with reports that passage of some intracellular bacteria via free living amoeba selects for virulence traits and enhances pathogenicity such as the ability to evade destruction in mammalian macrophages [16,20,33,34].

We examined the sections of the ulcers and observed the classical localized bed of acid fast bacilli at the undermined sections of the ulcer similar to that found in BU patients. Regular release of exudates, also characteristic of BU in humans, were also observed. Cultures of the lesion aspirates and lesion homogenates revealed the presence of viable *M*. *ulcerans* suggesting that the lesions seen were due to *M*. *ulcerans* infection. This study has therefore discovered the ICR mouse as a potential animal model for BU studies.

To summarize, we have demonstrated clearly that passive inoculation via punctured skin could be another route of transmission of BU. We have also identified the ICR mouse as another animal model for BU studies. In addition to the footpad, we have also identified the rump as another site for *M*. *ulcerans* inoculations, and which in addition to the known BU presentations in mice, also elicits undermined ulcer—the hallmark of BU. Finally, our results demonstrate for the first time the possible role of *A*. *polyphaga* in the transmission of BU by enhancing the pathogenicity of BU when cocultured with *M*. *ulcerans* and possibly playing the role of a vector.

## Supporting information

S1 Fig The progressive development of BU in ICR mouse topically treated with *M*. *ulcerans* at the punctured skin of the rump (lower back). Panel A shows site of inoculation 1 dpi. Panel B shows inflammation (erythema) at site of inoculation 31 dpi. Panel C shows an edema at site of inoculation 45 dpi. Panel D shows ulcer at the site of inoculation 49 dpi. Photograph is representative of group (n = 3).
(TIF)
