# Supplementary figures and images for: Experimental demonstration of the possible role of Acanthamoeba polyphaga in the infection and disease progression in Buruli Ulcer (BU) using ICR mice
Source: PLoS One. 2017 Mar 22;12(3):e0172843. doi: 10.1371/journal.pone.0172843 (PMC5362167; doi:10.1371/journal.pone.0172843)

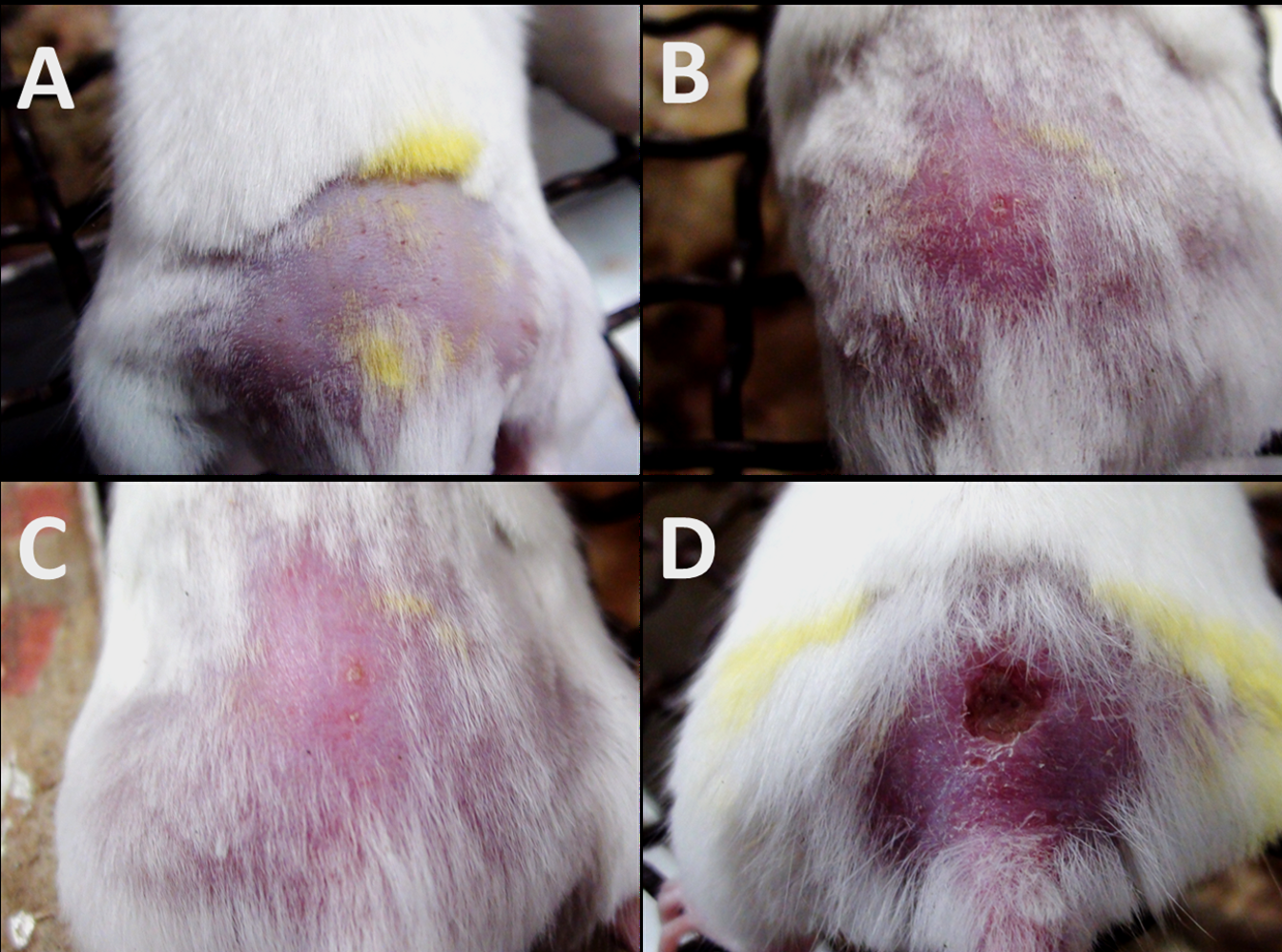

Supplement: S1 Fig — Panel A shows site of inoculation 1 dpi. Panel B shows inflammation (erythema) at site of inoculation 31 dpi. Panel C shows an edema at site of inoculation 45 dpi. Panel D shows ulcer at the site of inoculation 49 dpi. Photograph is representative of group (n = 3). (TIF) [file pone.0172843.s001.tif]
